# Extracellular Water and Blood Pressure in Adults with Growth Hormone (GH) Deficiency: A Genotype-Phenotype Association Study

**DOI:** 10.1371/journal.pone.0105754

**Published:** 2014-08-26

**Authors:** Edna J. L. Barbosa, Camilla A. M. Glad, Anna G. Nilsson, Niklas Bosaeus, Helena Filipsson Nyström, Per-Arne Svensson, Bengt-Åke Bengtsson, Staffan Nilsson, Ingvar Bosaeus, Cesar Luiz Boguszewski, Gudmundur Johannsson

**Affiliations:** 1 Department of Endocrinology, Institute of Medicine, Sahlgrenska Academy, University of Gothenburg, Gothenburg, Sweden; 2 SEMPR, Servico de Endocrinologia e Metabologia do Hospital de Clínicas da Universidade Federal do Paraná, Curitiba, Brazil; 3 Sahlgrenska Center for Cardiovascular and Metabolic Research, Department of Molecular and Clinical Medicine, Institute of Medicine, Sahlgrenska Academy, University of Gothenburg, Gothenburg, Sweden; 4 Institute of Mathematical Sciences, Department of Mathematical Statistics, Chalmers University of Technology, Chalmers, Gothenburg, Sweden; 5 Department of Clinical Nutrition Unit, Sahlgrenska University Hospital, Sahlgrenska Academy, University of Gothenburg, Gothenburg, Sweden; Rouen University Hospital, France

## Abstract

**Objectives:**

Growth hormone deficiency (GHD) in adults is associated with decreased extracellular water volume (ECW). In response to GH replacement therapy (GHRT), ECW increases and blood pressure (BP) reduces or remains unchanged. Our primary aim was to study the association between polymorphisms in genes related to renal tubular function with ECW and BP before and 1 year after GHRT. The ECW measures using bioimpedance analysis (BIA) and bioimpedance spectroscopy (BIS) were validated against a reference method, the sodium bromide dilution method (Br^−^).

**Design and Methods:**

Using a candidate gene approach, fifteen single-nucleotide polymorphisms (SNPs) in nine genes with known impact on renal tubular function (*AGT, SCNN1A, SCNN1G, SLC12A1, SLC12A3, KCNJ1, STK39, WNK1* and *CASR*) were genotyped and analyzed for associations with ECW and BP at baseline and with their changes after 1 year of GHRT in 311 adult GHD patients. ECW was measured with the Br^−^, BIA, and BIS.

**Results:**

Both BIA and BIS measurements demonstrated similar ECW results as the reference method. At baseline, after adjustment for sex and BMI, SNP rs2291340 in the *SLC12A1* gene was associated with ECW volume in GHD patients (*p* = 0.039). None of the SNPs influenced the ECW response to GHRT. One SNP in the *SLC12A3* gene (rs11643718; *p* = 0.024) and three SNPs in the *SCNN1G* gene [rs5723 (*p* = 0.02), rs5729 (*p* = 0.016) and rs13331086 (*p* = 0.035)] were associated with the inter-individual differences in BP levels at baseline. A polymorphism in the calcium-sensing receptor (*CASR*) gene (rs1965357) was associated with changes in systolic BP after GHRT (*p* = 0.036). None of these associations remained statistically significant when corrected for multiple testing.

**Conclusion:**

The BIA and BIS are as accurate as Br^−^ to measure ECW in GHD adults before and during GHRT. Our study provides the first evidence that individual polymorphisms may have clinically relevant effects on ECW and BP in GHD adults.

## Introduction

The inter-individual variability of extracellular water volume (ECW) is influenced by factors such as age, sex, race, weight and height [1]. The importance of genetic factors has been demonstrated in a twin study suggesting that approximately 68% of the variation in ECW is inherited [2].

Growth hormone (GH) influences electrolyte and water handling in humans and animals [3], [4]. GH and IGF-I receptors are expressed in renal tubules [5] and their effects in the distal nephron promote a transient retention of sodium and water, associated with a sustained increase in ECW [3]. GH deficient (GHD) adults have decreased ECW [6], which is restored by GH replacement therapy (GHRT) [7]. In addition, GHD in adults has been associated with increased blood pressure (BP) [8], [9], and GHRT has been shown to reduce systemic vascular resistance [10], [11], improve arterial compliance [12], and increase endothelium-dependent and -independent vasodilation [13]-[16]. A meta-analysis of 10 randomized controlled studies concluded that GHRT lowers diastolic BP (DBP), but not systolic blood pressure (SBP), in GHD adults [17].

The measurement of ECW is a useful tool to assess the response of GHRT in GHD adults because it reflects an important biological action of GH. ECW can be determined by different methods; the sodium bromide dilution (Br^−^) has been considered the reference method [18]. However, the technique is time-consuming, invasive, expensive and unavailable in most settings. In contrast, bioelectrical impedance analysis (BIA) at a frequency of 50 kHz and bioelectrical impedance spectroscopy (BIS) are easier, faster, non-invasive and less expensive methods for the measurement of ECW [19], [20].

In the present study, our primary aim was to investigate whether single nucleotide polymorphisms (SNPs) in genes known to influence BP and renal tubular function [21]-[35] might have an impact on the inter-individual differences in ECW and BP of untreated GHD adults and on the changes in these parameters 1 year after GHRT. The secondary aim was to evaluate whether the BIA and BIS methods were as accurate and reliable as the reference method Br^−^ to estimate ECW in GHD adults with and without GHRT.

## Patients and Methods

### Ethics statement

Written informed consent was obtained from all patients. The guardians for the two youngest patients who were seventeen years old when they started GHRT provided written informed consent for their GHRT data to be used for research purposes. When the present genetic study began, these two patients were twenty-seven and thirty-one years of age, and both provided informed written consent for their GHRT data to be used in this genotype-phenotype study. All data used in this study have been anonymized. This study was approved by the Ethics Committee in Gothenburg, Sweden, and performed in accordance with the Declaration of Helsinki.

### Patients

The patients enrolled in this study are part of a larger prospective longitudinal cohort of adults with hypopituitarism and GHD (n = 457) treated at the Sahlgrenska University Hospital, Gothenburg, Sweden. From this cohort of consecutive patients commencing GH replacement, we excluded patients who refused genetic testing (n = 51), with missing data during GHRT (n = 57), enrolled in another study (n = 25) and with compliance problems (n = 13). Therefore, a total of 311 GHD adults (181 men) with a mean age of 49.8 yr (range 17–77), who were eligible for GHRT, were selected for this study. The diagnosis of GHD was confirmed by using a GH stimulation test (77.2% insulin tolerance test, 6.1% GHRH-Arginine, and 3.2% GHRH–Pyridostigmine) or a low serum IGF-I concentration together with three or more pituitary hormone deficiencies (13.5%) [36]. Two hundred and seventy-nine patients had adult onset GHD (AO-GHD) and 32 had childhood onset (CO-GHD). None of the AO-GHD patients had previously received GHRT. CO-GHD subjects had previously received GH therapy, which was interrupted at least 4 years before they were retested for GHD in adulthood. Patients with previous treatment for Cushing's disease (n = 19), and acromegaly (n = 10) were in remission before entering the study and fulfilled international diagnostic criteria for GHD [36]. Overall, non-functioning pituitary adenoma was the most frequent aetiology of GHD (n = 131). When required, patients received adequate and stable replacement therapy with glucocorticoids, thyroid hormone, sex steroids and desmopressin for at least 6 months before starting GHRT. One hundred and fifty-six patients required hydrocortisone replacement therapy with a mean dose of 19.9±7.2 mg/day and 231 subjects required levothyroxine replacement therapy with a mean dose of 106.7±35.7 µg/day. Fifty women received oral (n = 37) or transdermal (n = 13) oestrogen therapy, while all hypogonadal men received testosterone by an intramuscular (n = 111) or transdermal (n = 23) route.

### Study design

Patients were prospectively enrolled in an open label treatment study. All patients received recombinant human GH, administered sc every evening, with an initial mean ± SD dose of 0.23±0.23 mg/day, which was titrated after 1 and 4 weeks and every 3 months subsequently to maintain age– and sex–adjusted serum IGF-I levels between the mean and the upper limit of the normal reference range.

### Anthropometry and blood pressure

Body weight (BW) was measured in the morning to the nearest 0.1 kg and body height was measured barefoot to the nearest 0.1 cm. The BMI was calculated as BW in kilograms divided by height in squared meters. SBP and DBP were measured after at least 5 min of supine rest using the sphygmomanometric cuff method.

### Biochemical assays

Serum IGF-I levels were determined in serum samples collected after an overnight fast, using a hydrochloric acid-ethanol extraction RIA with authentic serum IGF-I for labelling (Nichols Institute Diagnostics, San Juan Capistrano, CA, USA). After June 2004, the levels were determined using an automated chemiluminescent immunoassay (Advantage) from Nichols. From September 2006, serum IGF-I was determined using an automated chemiluminescent assay system (IMMULITE 2500, Diagnostic Products Corp., Los Angeles, CA, USA). All assays had a detection limit ≤20 µg/l and an inter-assay CV≤8.6%. The individual serum IGF-I levels were transformed into SD scores (SDS) according to age– and sex–adjusted reference values [37]-[38].

### Sodium bromide dilution method (Br^−^)

The ECW compartment was determined following the oral administration of sodium bromide (45 ml of a 5% NaBr solution) in a subset of 72 adults with GHD. The administered dose was 21.42 mmol of Br^−^ (corrected for 2% H_2_O). Blood samples were taken after 3 hours, and the Br^−^ was assayed in serum ultrafiltrate by high-performance liquid chromatography according to the method of Miller *et al.* [39]. The coefficient of variation (within patients) was 1.1%. The corrected bromide space (CBS) was calculated from the serum Br^−^ concentration after the administration of known amount of Br^−^.

The ECW was calculated as follows:
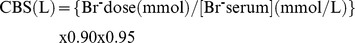
where 0.90 is the correction factor for the distribution of Br^−^ in the non-extracellular sites, and 0.95 is the Donnan equilibrium factor.

### BIA method (BIA)

With the patient in the supine position, BIA was measured with a frequency of 50 kHz using the BIA-101 device (RJL System, Detroit, MI, USA), according to the instructions of the manufacturer. ECW was calculated applying a formula for extrapolating the results in a subset of 80 GHD patients, as previously reported [19], [20]. To validate this formula, the ECW calculated by BIA was compared with ECW measured by the reference method (Br^−^) at baseline and 1 year after GHRT.

### BIS method (BIS)

ECW was measured with multifrequency BIS in a subset of 68 GHD patients. Xitron 4000B impedance analyzer (Xitron Technologies, Inc., San Diego, CA, USA) was used to measure resistance, reactance, impedance and phase angle at frequencies between 5 and 500 kHz. The calculations were previously described by De Lorenzo *et al.* [40] and Matthie *et al.* [41]. The coefficient of variation in duplicate samples in 43 patients was 0.3% [42]. ECW calculated by BIS was compared with ECW measured by the reference method (Br^−^) at baseline and 1 year after GHRT.

### Selection of candidate genes and SNPs

The selection of candidate genes was based on their physiological function in the regulation of the renal sodium and water balance according to previous publications. Further selection of candidate SNPs was based on earlier reports on functionality and/or associations with mean 24 hours BP [23], [34], [35], development of hypertension [22], [25]-[27], [30], [32], [33], [34], BP response to thiazide [24], [31], and renal dysfunction [21], [28], [32]. Due to the limited size of our cohort, SNPs with an allele frequency below 10% in the HapMap CEU Panel (when data were available in the Entrez SNP database) were not included.

The following SNPs in genes related to renal tubular function were studied: angiotensinogen (*AGT*, rs699) [21]; sodium channel, non-voltage-gated 1 alpha subunit (*SCNN1A*; rs2228576) [30]; sodium channel, non-voltage-gated 1 gamma subunit (*SCNN1G*; rs5723, rs5729, and rs13331086) [31]-[33]; solute carrier family 12 (sodium/potassium/chloride transporters), member 1 (*SLC12A1[NKCC2]*; rs2291340) [25]; solute carrier family 12 (sodium/chloride transporters), member 3 (thiazide-sensitive sodium-chloride cotransporter) (*SLC12A3*; rs11643718) [26]-[29]; potassium inwardly-rectifying channel, subfamily J, member 1 (*KCNJ1[ROMK]*; rs2186832, rs2846679, and rs675759) [35]; WNK lysine deficient protein kinase 1 (*WNK1*; rs880054, rs765250, and rs1159744) [23]-[24]; calcium-sensing receptor (*CASR*; rs1965357) [34]; serine threonine kinase 39 (*STK39*; rs3754777, and rs6749447) [22].

### Genetic analyses

Genomic DNA was isolated from whole blood using the Flexigene DNA kit (QIAGEN, Hilden, Germany). SNPs rs11643718 and rs3754777 were genotyped using TaqMan SNP genotyping and the remaining SNPs with the Sequenom platform.

In the TaqMan SNP genotyping, 10 ng genomic DNA was added to a reaction mix containing 1x TaqMan Genotyping PCR Master Mix (Applied Biosystems, Foster City, CA, USA) and SNP-specific genotyping assays purchased from Applied Biosystems (rs11643718, C__9609063_10, and rs3754777, C__27474774_10). All reactions were carried out in 5 µl reactions on 384-well plates (Applied Biosystems). PCR amplification was performed using a 384 dual GeneAMP PCR system 9700 instrument (Applied Biosystems) and allele detection was carried out in an ABI Prism 7900 HT Sequence Detection System instrument (Applied Biosystems).

The Sequenom genotyping was performed at the Mutation Analysis facility at Karolinska University hospital using matrix-assisted laser desorption/ionization time of flight (MALDI-TOF) mass spectrometry (Sequenom Inc., San Diego, CA, USA). iPLEX assays were designed using SpectroDESIGNER software (Sequenom Inc.). Amplification was performed in a total volume of 5 µl containing 10 ng of genomic DNA, 100 nM of each amplification primer, 500 mM of dNTP mix, 1.625 mM MgCl2 and 0.5 units of HotStarTaq DNA Polymerase (Qiagen Inc.). The reaction was subjected to the following PCR conditions: a single cycle of denaturation at 95°C for 15 min, followed by 45 cycles at 94°C for 20 s, 56°C for 30 s, 72°C for 60 s and a final extension at 72°C for 3 min. The allele-specific extension step was performed in a total volume of 9 µl using 5 pmol of extension primer and the Mass EXTEND Reagent Kit and cleaned using SpectroCleaner (Sequenom Inc., San Diego, CA, USA). Primer sequences are given in Table S1. Products from primer-extension reactions were loaded on a 384-element chip nanoliter pipetting system (Sequenom Inc.) and analyzed on a MassARRAY Compact mass spectrometer (Sequenom Inc.). The genotype calls were manually checked by two individuals separately using the SpectroTYPER RT 4.0.5 software (Sequenom Inc.). The genotyping was validated using a set of 14 trio families, totalling 42 individuals, with genotype data available through the HapMap consortium (HapMap data release 24/phase II).

### Statistical analysis

Statistical analysis was performed using SPSS for Windows, version 17.0 (SPSS Inc. Chicago, IL). For paired-samples, Student's *t* tests were used to compare values at baseline and after 1 year of GHRT. Agreement between methods of measuring ECW was performed using Bland-Altman analysis. The solid line represents the mean differences between the methods. The dashed lines represent the 95% limits of agreement (±1.96 SD).

Based on genotype, patients were divided into two groups: 1) patients with two major alleles and 2) patients carrying at least one minor allele. The genetic association of individual SNPs to the ECW and BP values at baseline as well as to their changes after 1 year of GHRT was analyzed with ANCOVA, adjusting for significant covariates. The covariates analyzed were age, sex, height, weight, BMI, testosterone replacement [43], dosage of glucocorticoids and thyroxine, use of anti-hypertensive drugs, prior treatment for Cushing's disease and acromegaly. *P*<0.05 was assumed to represent a significant difference. Only nominal *p*-values are presented in text and tables, but consideration of multiple testing was done using the Bonferroni method.

## Results

### Effects of GH replacement therapy

The mean daily GH dose after 1 year of GHRT was 0.4±0.2 mg. Mean serum concentration of IGF-I increased (from 107.5 µg/L at baseline to 252.8 µg/L after 1 year, *p*<0.0001). Changes in weight and BMI were not significant after GHRT. The mean increase in ECW was 0.5 L, as measured by BIA, and the mean decrease of SBP and DBP after GHRT was 1.7 mmHg (*p* = 0.045) and 1.5 mmHg (*p* = 0.007), respectively (Table 1).

**Table 1:** Effects of 1 year of growth hormone replacement therapy on clinical variables, IGF-I levels and extracellular water volume (ECW) assessed by bioimpedance (BIA).

	Baseline	1 year	Change	*P*-value
Weight (kg)	83.2±18.4	82.7±18.3	-0.5±6.3	0.177
BMI (kg/m^2^)	28.7±5.3	27.4±5.2	-1.3±2.1	0.07
SBP (mmHg)	129.4±18.5	127.6±17.7	-1.7±15.0	0.045
DBP (mmHg)	79.0±10.1	77.6±10.2	-1.5±9.4	0.007
IGF-I (µg/L)	107.5±69.3	252.8±122.7	145.2±100.8	<0.0001
IGF-I SDS	-1.2±1.2	0.9±2.5	2.1±2.3	<0.0001
ECW_BIA_ (L)	17.3±4.3	17.8±4.3	0.5±1.2	<0.0001

Data are presented as mean ± SD. Change (1 year minus baseline). BMI, body mass index. SBP: systolic blood pressure; DBP: diastolic blood pressure. SDS, standard deviation score.

### Accuracy of ECW methods

**Figure 1:**
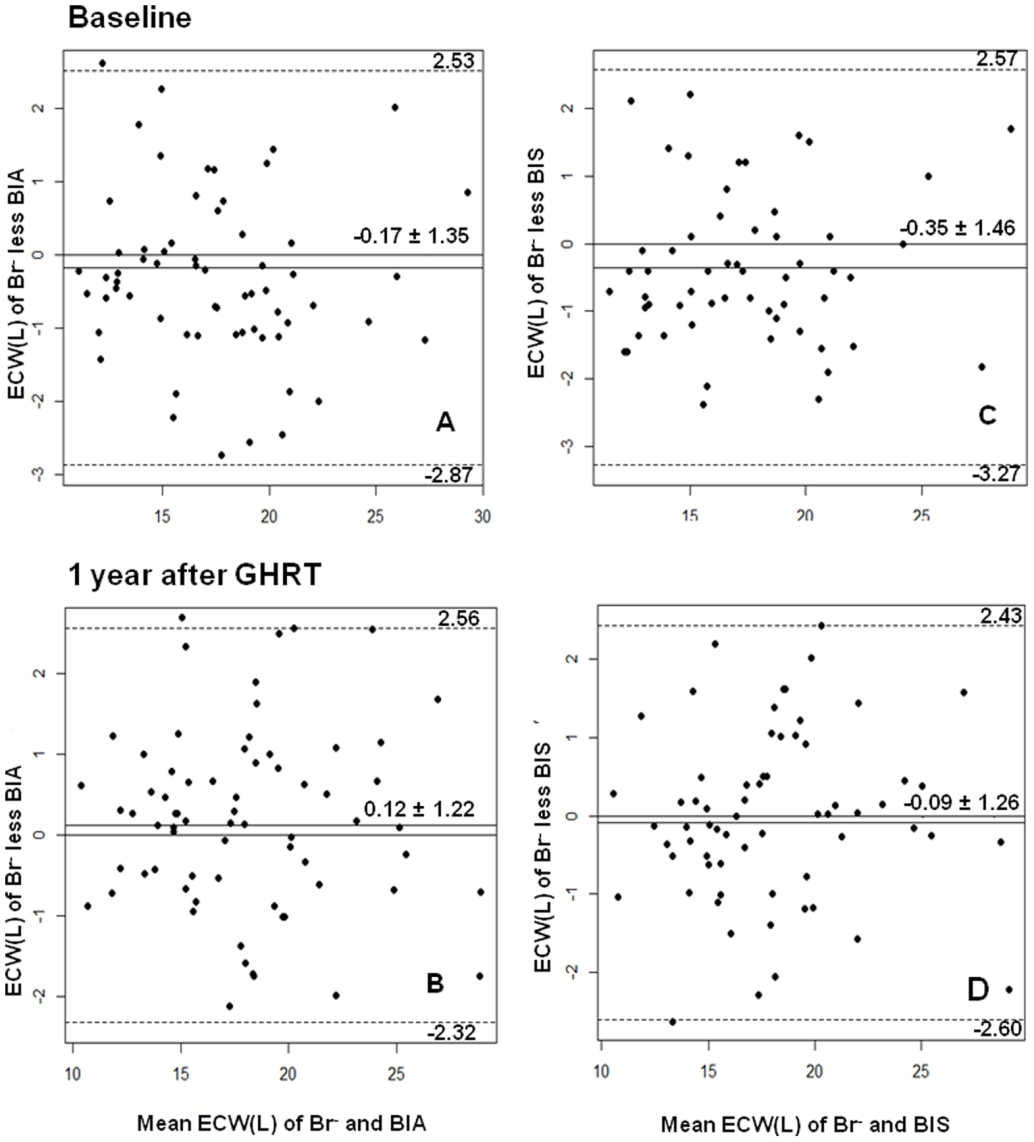
Agreement between methods using Bland-Altman analysis. The agreement between methods using Bland-Altman analysis for the measurements of extracellular water volume (ECW) by sodium bromide dilution method (Br^−^), single frequency bioelectrical impedance analysis (BIA) or bioelectrical impedance spectroscopy (BIS) before and after 1 year of growth hormone replacement therapy (GHRT). The solid line represents the mean differences between the methods and the dashed lines represent the lower and upper 95% limits of agreement (±1.96 SD) for the measurements. Br^−^ *vs* BIA (A and B) and Br^−^ *vs* BIS (*C and D*); before (*A and C*) and after GHRT (*B and D*).

ECW measurements assessed by Br^−^ were strongly correlated to the measurements obtained by BIA or by BIS before (r = 0.95, *p*<0.0001; and r = 0.93, *p*<0.0001, respectively) and after 1 year of GHRT (r = 0.96, *p*<0.0001; and r = 0.95, *p*<0.0001, respectively). There was also good agreement between Br^−^ and BIA (Figure 1A and Figure 1B) and between Br^−^ and BIS (Figure 1C and Figure 1D) by the Bland-Altman analysis, as the mean differences between ECW measured by the Br^−^ and BIA or BIS were close to zero, with no statistically significant difference in values obtained before and after GHRT. There were no differences in the change in ECW after 1 year of GHRT between the three different methods (Table 2).

**Table 2:** Changes in extracellular water volume (**Δ** ECW) after 1 year of growth hormone replacement therapy measured by the sodium bromide method (Br^−^), single frequency bioelectrical impedance analysis (BIA) or bioelectrical impedance spectroscopy (BIS).

	N	Baseline	1 year	Δ ECW	Mean difference (ΔECW_Br_^−^ - ΔECW_BIA or BIS_)
ECW_Br_^−^ (L)	56	17.5±4.1	17.9±4.4	0.5±1.7	0.2±1.6 (*p* = 0.32)
ECW_BIA_ (L)	56	17.6±4.2	17.9±4.4	0.3±1.0	
ECW_Br_^−^ (L)	49	17.2±3.7	17.7±4.1	0.5±1.7	0.2±1.8 (*p* = 0.42)
ECW_BIS_ (L)	49	17.6±3.9	17.9±4.0	0.3±1.1	

Data are presented as mean ± SD.

### Genotyping

All genotyping assays had a success rate >94.9%, except for rs2846679, which was excluded from the analysis (0% success rate). Additionally, after the Sequenom run, re-genotyping of 27% of the study samples resulted in 99.9% concordance. No Mendelian errors were found in the 14 HapMap families and concordance analyses with the HapMap data showed concordance rates of 100% for all analysed SNPs available in HapMap. No genotype data were available concerning rs880054 and rs1965357 from the HapMap consortium. Analysis of the parent-offspring-compatibility were performed on data from all SNPs and showed 100% compatibility for all of them. Minor allele frequencies (MAF), genotype distributions and Hardy-Weinberg equilibrium (HWE) *p*-values are shown in Table 3. Most of the polymorphisms that we studied had a MAF ≥10% and no deviation from the HWE (*p*>0.05). Only one SNP (rs11643718 in the *SLC12A3* gene) with a MAF of 9% was included in our analyses due to its clinical relevance. The *SLC12A3* gene encodes the thiazide-sensitive NaCl cotransporter, which is expressed in the distal convoluted tubule and plays an important role in renal electrolyte transport and BP maintenance [26]-[29].

**Table 3:** Minor allele frequencies, genotype distributions and concordance with Hardy-Weinberg equilibrium (HWE) of the 15 single-nucleotide polymorphisms (SNPs) in 9 genes related to renal tubular function and blood pressure in 311 growth hormone deficient adults.

Gene	SNP	Major(M)	Minor(m)	MAF(m)	MM	Mm	mm	missing	*P*-value (HWE)
*AGT*	rs699	A	G	0.44	101	144	64	2	0.63
*SCNN1A*	rs2228576	C	T	0.37	120	144	41	6	0.98
*SCNN1G*	rs5723	C	G	0.21	193	104	12	2	0.91
	rs5729	T	A	0.21	193	105	13	0	0.96
	rs13331086	T	G	0.22	185	105	13	8	0.93
*SLC12A1*	rs2291340	T	C	0.19	205	93	13	0	0.84
*SLC12A3*	rs11643718	G	A	0.09	256	51	2	2	0.95
*KCNJ1*	rs675759	G	C	0.17	220	79	12	0	0.36
	rs2186832	C	G	0.19	207	86	17	1	0.15
*STK39*	rs3754777	C	T	0.15	221	82	5	3	0.70
	rs6749447	T	G	0.24	168	124	12	7	0.17
*WNK1*	rs880054	T	C	0.38	124	137	48	2	0.61
	rs765250	T	C	0.24	179	112	20	0	0.91
	rs1159744	G	C	0.18	205	86	13	7	0.59
*CASR*	rs1965357	T	C	0.12	235	65	5	6	0.98

***AGT***, angiotensinogen gene; ***SCNN1A***, sodium channel, non-voltage-gated 1 alpha subunit; ***SCNN1G***, sodium channel, non-voltage-gated 1 gamma subunit; ***SLC12A1 [NKCC2]***, solute carrier family 12, member 1; ***SLC12A3***, solute carrier family 12, member 3; ***KCNJ1 [ROMK]***, potassium inwardly-rectifying channel, subfamily J, member 1; ***STK39***, serine threonine kinase 39; ***WNK1***, WNK lysine deficient protein kinase 1; ***CASR***, calcium-sensing receptor. **MAF**, minor allele frequency. **MM**, homozygote for the common (major) allele (two major alleles). **Mm**, heterozygote (one major allele, and one minor allele). **mm**, homozygote for the rare (minor) allele (two minor alleles).

### Genotype and ECW

The ECW volumes obtained using the BIA method was used in the association study between genetic polymorphisms and ECW. At baseline, the significant covariates for ECW were sex and BMI. After adjustment for confounders, the ECW of the homozygotes of the major allele of SNP rs2291340 in the *SLC12A1* gene was 0.6 L higher in comparison with the carriers of the minor allele (*p* = 0.039) (Table 4).

**Table 4:** Single nucleotide polymorphisms (SNPs) found to be associated with extracellular water volume (ECW), systolic (SBP) and diastolic blood pressure (DBP) in growth hormone deficient (GHD) adults at baseline and with their changes (**Δ**) after 1 year of GH replacement therapy (GHRT).

Outcome	Gene	SNP	MM (n)	Mm + mm (n)	Adjusted estimate	Adjusted *P-value
**Baseline**						
**ECW**	*SLC12A1*	rs2291340	TT (205)	TC + CC (106)		
			18.0±4.4	17.2±3.8	-0.6	0.039
**SBP**	*SLC12A3*	rs11643718	GG (252)	GA + AA (52)		
			129.6±18.7	127.1±17.6	-5.5	0.024
**DBP**	*SCNN1G*	rs5723	CC (190)	CG + GG (114)		
			80.0±10.3	77.5±9.5	-2.6	0.020
		rs5729	TT (190)	TA + AA (116)		
			80.0±10.3	77.5±9.4	-2.6	0.016
		rs13331086	TT (182)	TG + GG (116)		
			79.9±10.4	77.6±9.4	-2.3	0.035
**After GHRT**						
**ΔSBP**	*CASR*	rs1965357	TT (226)	TC + CC (70)		
			-2.8±15.1	1.6±14.5	-4.3	0.036
**ΔDBP**	*CASR*	rs1965357	TT (226)	TC + CC (70)		
			-2.0±8.9	0.5±10.6	-2.5	0.055

Data are presented as mean ± SD. Change (**Δ** = 1 year minus baseline). **MM**, carriers of two major alleles. **Mm + mm**, carriers of at least one minor allele. **Adjusted estimate** and ***P*** **-value** are the estimated difference between genotype groups in ANCOVA, after adjustment for the significant covariates: sex and BMI for ECW and age and BMI for BP.

Additional analyses excluding patients receiving anti-hypertensive drugs (n = 67), patients with previous Cushing's disease (n = 19) and acromegaly (n = 10) did not alter the results.

No single SNP was significantly correlated with the ECW changes induced by GHRT.

### Genotype and blood pressure

Table 4 summarizes the SNPs significantly associated with BP at baseline and BP changes after 1 year of GHRT.

At baseline, after adjustment for the significant covariates age and BMI, SNP rs11643718 in the *SLC12A3* gene was associated with SBP, while SNPs rs5723, rs5729, and rs13331086 in the *SCNN1G* gene were associated with DBP. The mean SBP of carriers of the minor allele of SNP rs11643718 in the *SLC12A3* gene was 5.5 mmHg lower in comparison with the SBP of the homozygotes of the major allele. The mean DBP of carriers of the minor allele of the SNPs rs5723, rs5729 and rs13331086 in the *SCNN1G* gene was 2.6, 2.6, and 2.3 mmHg, respectively, lower than that of the homozygotes of the major allele.

Additional analyses excluding patients receiving anti-hypertensive drugs (n = 67), patients with previous Cushing's disease (n = 19) and acromegaly (n = 10) did not alter these results.

**Figure 2:**
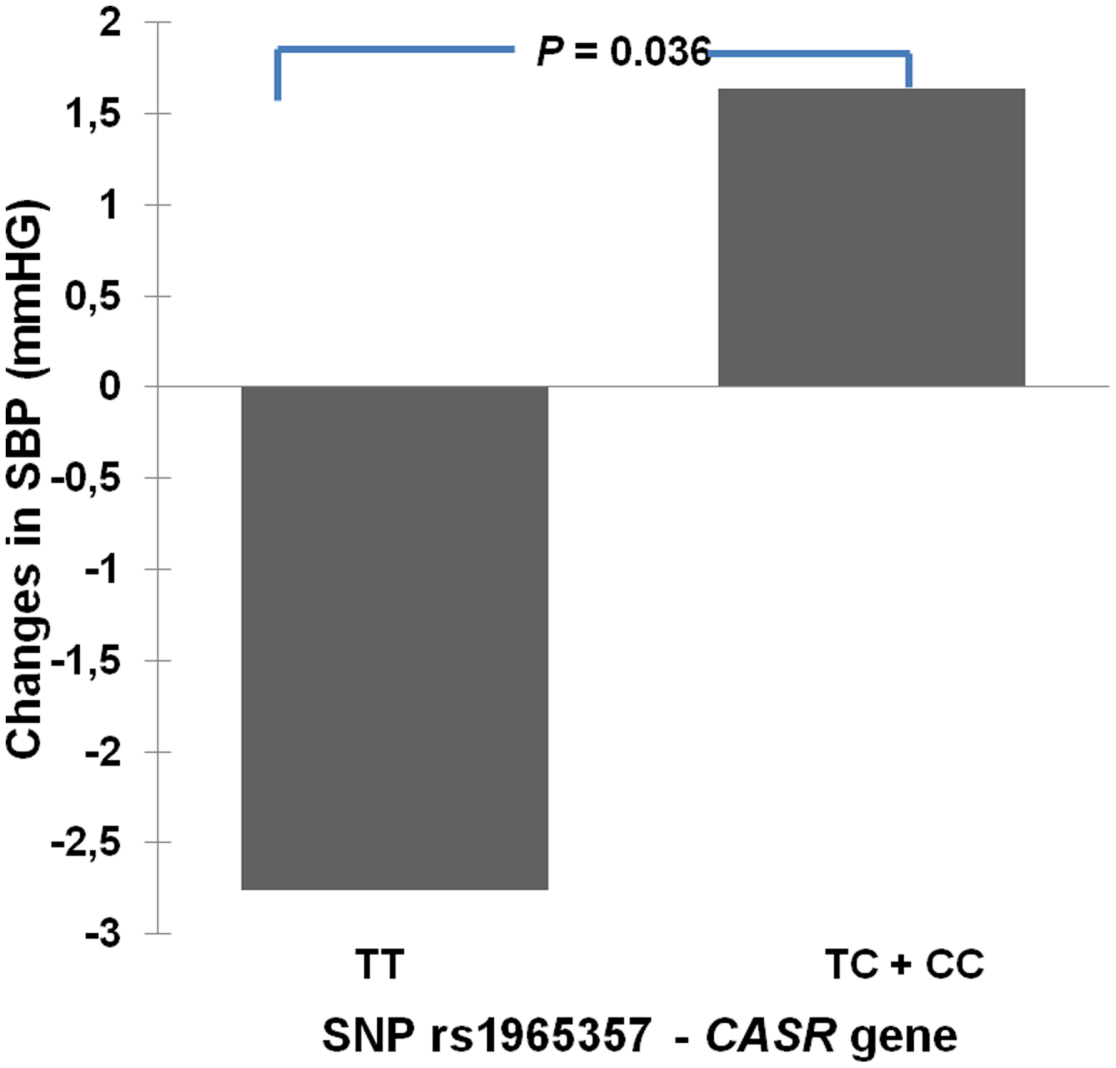
Association of the Calcium-Sensing Receptor gene with changes in systolic blood pressure after GH therapy. Influence of the single nucleotide polymorphism (SNP) rs1965357 in the Calcium-Sensing Receptor gene (*CASR*) gene on the changes in systolic blood pressure (SBP) after 1 year of growth hormone replacement therapy (GHRT) in GH deficient adults. TT: carriers of two major alleles; TC + CC: carriers of at least one minor allele.

After 1 year of GHRT, after adjustment for age and BMI, SNP rs1965357 in the *CASR* gene was associated with changes in SBP (*p* = 0.036) and there was a trend for association between this SNP and the changes in DBP (*p* = 0.055). The mean reduction in SBP (Table 4, Figure 2) and DBP (Table 4) during treatment of homozygotes of the major allele was larger than in those carriers of the minor allele of SNP rs1965357. Additional analyses excluding patients receiving anti-hypertensive drugs (n = 67), patients with previous Cushing's disease (n = 19) and acromegaly (n = 10) did not alter these results.

The associations did not remain significant when we performed correction for multiple testing (Bonferroni correction).

## Discussion

We found that the BIA and BIS methods were as reliable as the reference method (Br^−^) for measuring ECW in GHD adult patients allowing us to use these results in the genotype-phenotype association study. We evaluated 15 SNPs from nine candidate genes in relation to both ECW and BP levels before and after GHRT. We found that one SNP in a gene related to renal sodium and water balance (*SLC12A1* rs2291340) had a significant association with ECW of untreated GHD adults. Moreover, one SNP in the *SLC12A3* gene (rs11643718) was associated with SBP and three SNPs in the *SCNN1G* gene (rs5723, rs5729, rs13331086) were associated with DBP levels in untreated GHD adults. The change in SBP in response to GHRT was associated with the SNP rs1965357 in the *CASR* gene.

It is well established that genetic factors influence the ECW content [2]. Therefore, we conducted a candidate gene approach by selecting 15 SNPs in genes which were previously associated with renal sodium and water balance functionality and/or with BP variation [22], [23], [25]-[27], [29]-[35], and renal dysfunction [21], [28], [34]. These polymorphisms were then related to ECW and BP before and after GHRT. Interestingly, the observed effect of genotypes on baseline ECW was of the same magnitude (0.5 to 0.6 L) as the increase in ECW after GHRT. These results indicate that individual SNPs may have clinically relevant effects on ECW in GHD adults.

We found a nominal association dependent on sex and BMI between SNP rs2291340 in the *SLC12A1* gene and ECW at baseline. This SNP was selected for our study because its contribution to BP variation was previously reported [25]. The *SLC12A1* gene encodes a kidney-specific sodium-potassium-chloride cotransporter (Na-K-Cl cotransporter) that is expressed on the luminal membrane of renal epithelial cells of the thick ascending limb of Henle's loop and the macula densa. It plays a key role in concentrating urine and accounts for most of the NaCl reabsorption. Na-K-Cl cotransporter activity is affected by a large variety of hormonal stimuli, including GH [44]. GH acutely increases renal electrolyte and water reabsorption by modulating the Na-K-Cl cotransporter in distal tubular segments.

Genes related to renal tubular functions have been shown to be associated with BP. One example is the *CASR* gene, which encodes a G protein-coupled receptor that is expressed in the cells lining the kidney tubules. In thick ascending limb, the CaSR protein is localized on basolateral cell membranes and inhibits the Na, K, 2Cl cotransporter, which decreases sodium reabsorption and, secondarily, decreases Ca reabsorption. *CASR* gene variants could influence BP by affecting sodium retention. In a study of normotensive African-Americans, common variations in *CASR* gene including SNP rs1965357 have been associated with individual differences in BP and with urinary excretion rates of Ca in both population-based and family-based analyses (34). In our cohort, we found significant associations of this SNP in the *CASR* gene with changes in SBP and DBP levels after 1 year of GHRT. These findings suggest that individual differences in BP response in GHD adults on GHRT could be related to CaSR-mediated influences on sodium uptake in thick ascending limb.

The thiazide-sensitive NaCl cotransporter (*NCC*, also known as *SLC12A3*) mediates salt reabsorption in the distal nephron of the kidney and is the target of thiazide diuretics. Mutations in *SLC12A3* give rise to Gitelman's syndrome, a hereditary salt-wasting disorder [45]. A polymorphism of the NCC-coding gene *SLC12A3* (rs133066739) has been associated with the response to thiazide diuretics [46]. Two studies in hypertensive patients where Arg904 was replaced with Gln in this gene found that minor allele carriers were predisposed to primary hypertension (26-27). In a large cohort of patients with type 2 diabetes mellitus the *SLC12A3* gene was identified as a candidate gene for conferring susceptibility to diabetic nephropathy, and that the substitution of Arg913 to Gln might be associated with reduced progression of nephropathy (28). Although the exact mechanism for this reduced risk in patients with the minor allele is not yet clear, the loss of function of this thiazide-sensitive co-transporter has been reported as significantly reducing arterial blood pressure in sodium-restricted mice (29). Consequently, it may be assumed that substituting Gln for Arg913 could affect the blood pressure of humans under pathological conditions such as diabetes mellitus. This is in line with the present study, where the SNP rs11643718 (also known as Arg913Gln) was found to be associated with SBP variation in untreated GHD adults, with lower systolic pressure in minor allele carriers.

The epithelial sodium channel (ENaC) plays a vital role in BP regulation. Mutations in the gene encoding the γ-subunit of ENaC, *SCNN1G*, were identified as causes of Mendelian forms of hypertension and hypotension [32]. Polymorphisms in this gene are associated with variations in BP [31]-[33]. Moreover, it was also significantly associated with BP in a metaanalysis of four cohorts with 1611 white European families, where the presence of the minor allele of this SNP (G) was associated with a 1.01 mmHg increase in SBP and 0.52 mmHg increase in DBP [33]. In the present study, the *SCNN1G* variants were associated with the inter-individual DBP variation in GHD adults. The minor alleles of the SNPs rs5723 (G), rs5729 (A), and rs13331086 (G) were associated with lower DBP levels at baseline.

BIA methods for measuring body fluids are noninvasive, rapid and simple procedures. However, they are indirect tools based on the difference in conduction of an electrical current through various body constituents, and their accuracy is very much dependent upon the validity of the tissue electrical model [47], [48]. The ECW and TBW estimates by single frequency BIA is based on empirical equations of the wrist-ankle resistance or impedance at 50 kHz, height and weight. It works well in healthy subjects and in patients with stable water and electrolytes balance with a validated BIA equation adjusted to age, sex and race [47]-[49]. However, the BIA values in GHD patients on GHRT must be interpreted with caution, as these patients have reduced ECW which are normalized with therapy, and the detection of these changes depends on the different regression equations applied [49], [50]. In our study, we used the method proposed by Jaffrin *et al.* [19], [20], which combine the single frequency BIA method with BIS equations. In the modification proposed by these authors [19], [20], and tested in healthy volunteers, values of resistance at zero (*R_e_*) and infinite frequencies (*R∞*) were determined from the resistance at 50 kHz (*R_50_*). Due to the strong correlations among these three resistances, errors made when *R_e_* and *R∞* are determined from *R_50_* are small and do not compromise the accuracy of the method. We did not observe significant differences between the Br^−^ and BIA results, suggesting that BIA can be as accurate as Br^−^ to determine ECW in GHD patients either on or off GHRT. Nevertheless, some discrepancies may occur for individual patients.

The multiple frequency BIS method estimates both ECW and ICW volumes. It relies on an electrical model based on resistances measured at zero and infinite frequencies in tissues [19], [20]. The method is useful to assess ECW in healthy subjects and in critically ill patients [51]. Similarly to what we observed with BIA, the ECW estimates by BIS and Br^−^ were highly comparable, showing that this method can also be employed to determine ECW in GHD adults.

There are obvious limitations of our study. Sample size is always a major concern in genotype-phenotype association studies, and although we have evaluated an impressive cohort of GHD adults prospectively followed in a single center, the experience shows that larger populations allow better power for data interpretation. This is exemplified by the lack of significance of our genotype-phenotype associations when we performed correction for multiple testing. In addition, the candidate gene approach does not exclude the possibility that other genes may be of greater importance. On the other hand, the design allows a targeted statistical approach, i.e. hypothesis generated, and therefore the findings cannot be summarily invalidated.

To conclude, this study is the first to evaluate the influence of common variants in genes related to the renal sodium and water balance on ECW and BP in GHD adults before and after GHRT. We were able to show that five polymorphisms in three genes were associated with ECW and BP in untreated GHD adults, while one polymorphism in another gene was associated with changes in systolic BP during therapy. Finally, we demonstrated that BIA and BIS are accurate methods to estimate ECW in GHD adults and to monitor ECW changes in response to GHRT.

## Supporting Information

Table S1 **Description of dbSNP ID and extension primer sequences used in the Sequenom genotyping experiment.** Primer sequences used for the Sequenom genotyping. Lower case letters indicate unspecific sequences added to the primers in order to receive a range of product masses.
(DOC)
