# Supplementary material for: Extracellular Water and Blood Pressure in Adults with Growth Hormone (GH) Deficiency: A Genotype-Phenotype Association Study
Source: PLoS One. 2014 Aug 26;9(8):e105754. doi: 10.1371/journal.pone.0105754 (PMC4144955; doi:10.1371/journal.pone.0105754)
Supplement: Table S1 — Description of dbSNP ID and extension primer sequences used in the Sequenom genotyping experiment. Primer sequences used for the Sequenom genotyping. Lower case letters indicate unspecific sequences added to the primers in order to receive a range of product masses. (DOC) [file pone.0105754.s001.doc]

**Table S1.** Description of dbSNP ID and

extension primer sequences used in the Sequenom

genotyping experiment

| **dbSNP ID** | **primer sequence** |
| --- | --- |
| rs699 | AAGACTGGCTGCTCCCTGA |
| rs5723 | TTCTCAACCCTGCCTCA |
| rs5729 | GGTGGAGATCAGAGTGCCG |
| rs13331086 | GCTGTGTCCGCATAGACT |
| rs2228576 | GGGCCCCCCCAGAGGACAGG |
| rs675759 | GAAAAAAACTGGTACTCAAAG |
| rs2186832 | CCTGTTTCAGAGCTCCACTTCATC |
| rs2291340 | AAGGAGGAAATTGGCATAAAAA |
| rs6749447 | ggGTCTGCTAGTACTAGATTAGGA |
| rs765250 | AAAAAGACATGCTCACCAAAACA |
| rs880054 | CTGCTCTCCTTTCCATATT |
| rs1159744 | gtgACTGATATTCTCTATTTGTTGAG |
| rs1965357 | TAGCTTCAGCTTTGTTTGTAA |

Primer sequences used for the Sequenom genotyping.

Lower case letters indicate unspecific sequences added

to the primers in order to receive a range of product masses.
